# Delayed Presentation of DPD Deficiency in Colorectal Cancer

**DOI:** 10.6004/jadpro.2014.5.3.5

**Published:** 2014-05-01

**Authors:** Lindsey Law, Jane Rogers, Cathy Eng

**Affiliations:** University of Texas MD Anderson Cancer Center, Houston, Texas

## Abstract

**Case Study**

Mr. D., a 55-year-old male, presented to the medical oncology service with a diagnosis of stage III adenocarcinoma of the sigmoid colon. He presented 7 weeks post sigmoid colectomy with lymph node resection and was initiated on adjuvant chemotherapy with CAPOX (capecitabine [Xeloda] and oxaliplatin [Eloxatin]). Standard dosing was used: oxaliplatin at 130 mg/m^2^ on day 1 and capecitabine at approximately 2,000 mg/m^2^/day (rounded to the nearest 500-mg tablet size) for 14 days on and 7 days off (1 cycle = 21 days). A capped body surface area of 2.4 m2 was used, due to the patient’s body habitus.

**Adverse Effects**

Mr. D. did not report any complications of therapy during cycle 1, days 1–7, other than grade 1 diarrhea, which was amenable to diphenoxylate/atropine when taken. The next week, he reported significant malaise and fatigue associated with persistent diarrhea occurring every 30 minutes for 5 days. Mr. D. was instructed to go to the emergency room for an immediate evaluation, but he refused.

Mr. D. presented to the clinic in poor condition on day 14 of cycle 1. His diarrhea had increased to grade 3 and was not controlled with either loperamide or diphenoxylate/atropine, though he was not taking his medications as directed. He had been instructed to take two 2-mg loperamide tablets after the first loose stool, followed by 1 tablet of diphenoxylate/atropine 2 hours later. He could then alternate this with loperamide every 2 hours as needed, not to exceed 8 tablets of loperamide per day. Instead, he had taken 2 tablets of loperamide after the first loose stool, but either waited 6 hours to take 1 tablet of diphenoxylate/atropine or otherwise chose not to alternate the medications at all despite continued diarrhea, depending on the day.

Mr. D.’s timing in taking his supportive medications was inconsistent, and his explanations of this timing were not exact. He also reported persistent grade 3 nausea with vomiting for 5 days, which did not improve with ondansetron and prochlorperazine, though he again did not take these consistently. He was advised to alternate ondansetron and prochlorperazine every 4 hours as needed, but only took one or the other medication approximately 3 times per day.

According to Mr. D., his adverse effects initially began on day 9 of cycle 1. He had lost approximately 14 kg (31 lb) during cycle 1. Clinically, he was found to have grade 2 mucositis and grade 1 hand-foot syndrome. At the time of this visit, his absolute neutrophil count was 3,000/ìL, his hemoglobin was 14.4 g/dL, his hematocrit 42.2%, and his platelet count was 139,000/ìL. His kidney function was within the normal range.

Mr. D. refused hospitalization despite the primary team’s recommendation. He also refused to undergo stool sampling for *Clostridium difficile*. He was given IV fluids along with adjustments in supportive medications, including a prescription for 10% tincture of opium. He was instructed to use 0.6 mL every 6 hours in addition to alternating loperamide with diphenoxylate/atropine as noted previously. He was advised to rinse his mouth with a baking soda solution for relief of his grade 1 mucositis, and alternation of antiemetics every 4 hours was reiterated. He was to return prior to initiation of cycle 2 for further evaluation.

**Worsening Symptoms**

The next day, Mr. D.’s wife called the clinic to report that her husband’s diarrhea continued despite the use of tincture of opium and that it was associated with hematochezia. He was also experiencing a worsening of his mucositis, with an associated swelling of the tongue. He was instructed to present to the emergency center, which he did on day 16 of cycle 1. By then, he was found to be febrile at 39.5°C. He was tachycardic, with a heart rate of 126, and he was experiencing significant abdominal pain associated with the diarrhea. The mucositis was worsening, with new odynophagia.

At this time, Mr. D.’s absolute neutrophil count had dropped dramatically to 160/ìL, his hemoglobin was 13.1 g/dL, his hematocrit was 39.2%, and his platelet count was 68,000/ìL. He was admitted to the inpatient service and started on empiric antibiotics. His blood cultures remained negative during hospitalization, but stool cultures were positive for *C. difficile*. His antimicrobial regimen was deescalated to oral vancomycin once his stool volume decreased. He was treated with an institutional compounded mouthwash of diphenhydramine, aluminum/magnesium hydroxide, and viscous lidocaine for the mucositis, which also slowly improved. He was given a dose of growth factor. Neutropenia eventually resolved, with an absolute neutrophil count of 4,820/ìL on the day of discharge. He was discharged 26 days after initiating cycle 1, at which time his myelosuppression and mucositis were also resolved. Throughout his course, he did not report any neurotoxicity.

**DPD Testing**

Due to his severe symptoms of neutropenia, mucositis, and diarrhea, Mr. D. was tested for dihydropyrimidine dehydrogenase (DPD) deficiency. Testing confirmed a heterozygous IVS14+IG>A mutation. For this reason, all further adjuvant therapy was withheld, and he was followed on clinical surveillance only.

Fluoropyrimidines such as fluorouracil (5-FU) and capecitabine are commonly prescribed agents for the management of gastrointestinal, breast, genitourinary, and head and neck cancers, with millions of patients receiving them each year (Ezzeldin & Diasio, 2004; Mercier & Ciccolini, 2006). While these drugs are generally well tolerated at standard doses, studies have shown that approximately 31% to 34% of treated cancer patients develop severe, dose-limiting toxicities. This is in part due to the narrow therapeutic window of 5-FU, combined with high interpatient pharmacokinetic variability, different dosing strategies, and enzyme deficiency (Mercier & Ciccolini, 2006). See Table 1 for common grade 3/4 toxicities seen in patients receiving capecitabine and 5- FU.

**Table 1:**
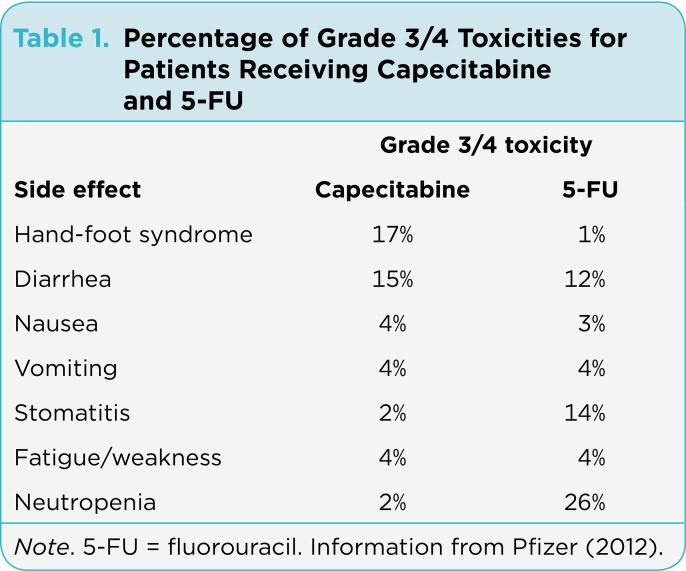
Table 1. Percentage of Grade 3/4 Toxicities for Patients Receiving Capecitabine and 5-FU

5-FU’s metabolism is a complex multienzymatic pathway. More than 85% of an administered dose is rendered inactivated by the rate-limiting enzyme DPD, leaving 15% for conversion to active metabolites, which leads to inhibition of DNA synthesis (Ezzeldin & Diasio, 2004; Mercier & Ciccolini, 2006; Saif et al., 2007). Capecitabine, an oral 5-FU prodrug, is commonly used in place of 5-FU due to the convenience of oral administration (Hoff et al., 2001; Twelves et al., 2001). It undergoes a three-step enzymatic conversion to 5-FU for its cytotoxic effects. Given that this agent is a prodrug to 5-FU, DPD remains an important component of capecitabine elimination.

##  DPD Deficiency

DPD deficiency is a pharmacogenetic syndrome caused by molecular defects or mutations in the *DPYD* gene that result in complete or partial loss of DPD enzyme activity (Ezzeldin & Diasio, 2004). Partial DPD deficiency is present in approximately 3% to 5% of adult cancer patients, with complete deficiency occurring in 0.5% (Mercier & Ciccolini, 2006). A deficiency in DPD leads to a shift to active 5-FU metabolites, an increase in the elimination half-life, prolonged exposure, and therefore significant plasma overexposure in patients treated with the standard doses of 5-FU (Ciccolini et al., 2006; Ezzeldin & Diasio, 2004). Given the already narrow therapeutic window of 5-FU, DPD deficiency results in exaggerated 5-FU–related toxicities, including neutropenia, mucositis, stomatitis, diarrhea, skin rash, neurologic toxicities, and even death (Ciccolini et al., 2006; Saif et al., 2007). It has been shown that 40% to 50% of patients with grade 3/4 toxicity to 5-FU displayed partial or complete DPD deficiency (Ezzeldin & Diasio, 2004).

Literature regarding DPD deficiency in the 5-FU setting is abundant. Less is known about its presentation with capecitabine administration. It is evident that subtle differences exist between 5-FU and capecitabine with regard to drug interactions and the incidence and severity of common adverse effects (Hoff et al., 2001; Twelves et al., 2001). Therefore, it is important to determine whether differences exist between 5-FU and capecitabine in the presentation of DPD-deficient patients. In the case report at the beginning of this article, we presented Mr. D., who had a delayed occurrence of capecitabine toxicity and was identified as having a DPD deficiency.

## Discussion

Given the widespread use of 5-FU and capecitabine for the treatment of GI malignancies, detection of DPD deficiencies with simple, rapid, and cost-effective screening methods is necessary. Despite the severity of toxicity associated with DPD deficiency, there has been no method for routine screening considered suitable due to technical limitations, wide range of bias, time, availability, and expense (Mercier & Ciccolini, 2006). The difficulty lies in the complexity of the *DPYD* gene and its high number of polymorphisms. There are over 40 mutations identified in the gene so far, though many have little or no obvious functional effect; this limits the usefulness of single-mutation genotyping (Ezzeldin & Diasio, 2004).

The patient in this case was positive for one copy of the DPYD*2A (IVS14+1G>A) mutation, the most frequently detected mutation associated with DPD deficiency (Ezzeldin & Diasio, 2004). This is a single-nucleotide polymorphism characterized by a G- to-A mutation in the 5ˇ splicing recognition sequence on intron 14 usually associated with the most severe reported 5-FU toxicities (Ciccolini et al., 2006; Ezzeldin & Diasio, 2004; Mercier & Ciccolini, 2006). There are several other deletions, missense mutations, point mutations, and even methylation of the DPYD promoter, which have all been related to DPD deficiency (Mercier & Ciccolini, 2006).

Select representative DPD deficiency cases within the capecitabine and 5-FU settings are summarized in Table 2. The first case in the table represents the case of Mr. D. presented here. In addition to our case, one other case of a DPD-deficient patient receiving CAPOX has been reported. However, unlike Mr. D., that patient did not recover from the toxicity. It is interesting to note that this toxic death case reported after administration of CAPOX was not associated with the most common DPYD*2A mutation, but with a heterozygosity for the 1896C>T mutation also located in exon 14 of the gene (Ciccolini et al., 2006; Mercier & Ciccolini, 2006).

**Table 2:**
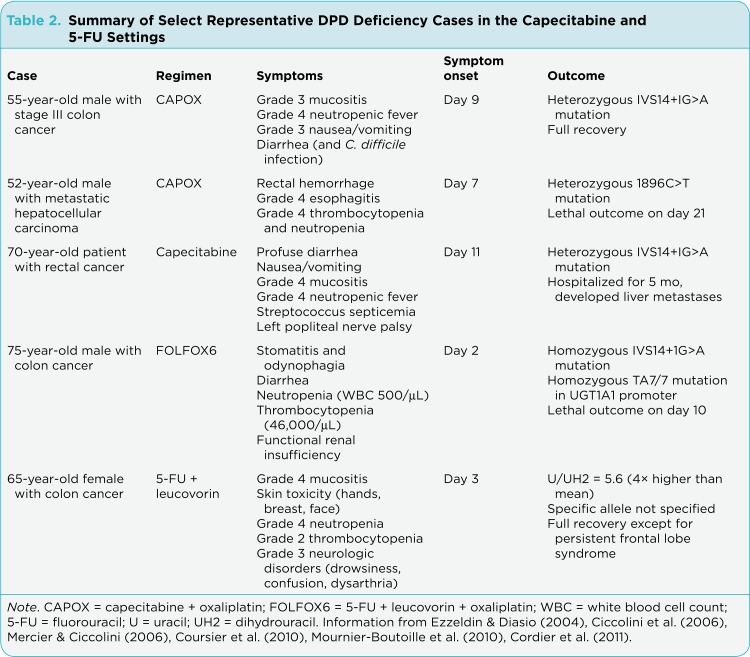
Table 2. Summary of Select Representative DPD Deficiency Cases in the Capecitabine and 5-FU Settings

Despite this difference, the two DPD-deficient patients described in the CAPOX cases presented with relative similarity in that they both reported initiation of severe toxicities around days 7–9, an atypical delayed reaction based on what has been reported with infusional 5-FU (Ciccolini et al., 2006; Cordier et al., 2011; Coursier et al., 2010; Mournier-Boutoille et al., 2010). For example, an additional case report involving the use of capecitabine in a DPD-deficient patient indicated that the patient was admitted to the ICU on day 11 after initiation of capecitabine (Coursier et al., 2010).

By contrast, a case of DPD deficiency in a metastatic colorectal cancer patient receiving 5-FU with oxaliplatin (FOLFOX) reported that the patient developed severe mucositis and odynophagia on day 2 after treatment (Mournier-Boutoille et al., 2010). He had an IVS14+1G>A homozygous mutation as well as a TA7/7homozygote mutation in the UGT1A1 gene promoter. Another colorectal cancer patient found to be DPD deficient was reported to have developed severe skin toxicities on day 3 after treatment with 5-FU and leucovorin and eventually developed neurotoxicity (Cordier et al., 2011).

All of the previously referenced cases of DPD deficiency appear to have considerably less time to toxicity compared to those without a DPD mutation according to the capecitabine package insert, which reports that the median time to first occurrence of grade 2–4 diarrhea is 34 days, and the median time to onset of hand-foot syndrome is 79 days (Pfizer, 2012). While there does not appear to be any standard time to toxicity onset for DPD-deficient patients, review of the above-mentioned cases raises the possibility that the reaction may be delayed for patients taking capecitabine vs. those taking infusional 5-FU.

Further, onset and severity in toxicity may differ, dependent on the specific mutation and the degree of mutation. For example, mutations in other alleles can yield similar (if not worse) outcomes than were seen in our case. This suggests that although it is the most common deficiency allele, the canonical IVS14+1G>A mutation might not be a reliable predictive marker on its own (Mercier & Ciccolini, 2006). It is also probable that the timing and severity of symptoms are related in part to the heterogeneity or severity of the individual polymorphism. All cases suggest that clinical suspicion for DPD deficiency should remain high for patients who develop severe toxicities independent of the timing of symptom onset and raise again the question of need for screening prior to starting a patient on a fluoropyrimidine.

## Screening

While there are numerous methods available to determine DPD status—including mass spectrometry, thin layer chromatography, and the gold standard radioenzymatic assay to name a few—many are expensive, time-consuming, or prone to ambiguous results. However, the recent development of a rapid (< 90 minutes), noninvasive, and cost-effective breath test in a clinical laboratory setting may permit the evaluation of DPD activity before the administration of 5-FU (Ezzeldin & Diasio, 2004; Mercier & Ciccolini, 2006). Another approach could be denaturing high-performance liquid chromatography (DHPLC), which can screen for both known and unknown sequence variations, as the entire *DPYD* gene can be scanned in 12.5 hours (Ezzeldin & Diasio, 2004). Currently, these two methods have not been utilized in a standard fashion but may allow expedited determination of DPD deficiency.

If a patient is determined to be positive for DPD deficiency, accepted alternative treatments are sought to avoid any unnecessary toxicity. In our case, for the patient seeking adjuvant chemotherapy for locally advanced colorectal cancer, the risks and benefits of additional 5-FU–based therapy must be discussed with the patient. Due to the severity of Mr. D.’s toxicity, he opted for close observation only. Hence, he was strongly urged to be adherent with all future surveillance visits. Because of the likelihood of distant disease recurrence due to his degree of nodal involvement, we opted to pursue biannual CT scans rather than an annual CT scan. In the metastatic colorectal cancer patient for whom chemotherapy is warranted, rather than pursuing 5-FU–based therapy, an alternative regimen such as irinotecan plus oxaliplatin (IROX) would be considered (Haller et al., 2008); irinotecan alone as cytotoxic chemotherapy in combination with a biologic targeted agent would be considered as well.

Though there is no method of testing that meets the criteria for standardized screening, we suggest that any method of testing is better than "blind administration of standard dosages of 5-FU performed regardless of the DPD status of patients with cancer," given the countless reports demonstrating the relationship between DPD deficiency and 5-FU/capecitabine-related toxicities and death (Mercier & Ciccolini, 2006).

## Conclusion

Advanced practitioners (APs) in oncology who administer 5-FU or capecitabine in any disease state must maintain suspicion for DPD deficiency in patients who develop severe toxicity regardless of symptom timing. It is imperative that APs manage patient symptoms through aggressive supportive care in hopes of curbing significant adverse effects. 
